# Figure Text Extraction in Biomedical Literature

**DOI:** 10.1371/journal.pone.0015338

**Published:** 2011-01-13

**Authors:** Daehyun Kim, Hong Yu

**Affiliations:** Department of Health Science, University of Wisconsin-Milwaukee, Milwaukee, Wisconsin, United States of America; University of South Florida College of Medicine, United States of America

## Abstract

**Background:**

Figures are ubiquitous in biomedical full-text articles, and they represent important biomedical knowledge. However, the sheer volume of biomedical publications has made it necessary to develop computational approaches for accessing figures. Therefore, we are developing the Biomedical Figure Search engine (http://figuresearch.askHERMES.org) to allow bioscientists to access figures efficiently. Since text frequently appears in figures, automatically extracting such text may assist the task of mining information from figures. Little research, however, has been conducted exploring text extraction from biomedical figures.

**Methodology:**

We first evaluated an off-the-shelf Optical Character Recognition (OCR) tool on its ability to extract text from figures appearing in biomedical full-text articles. We then developed a Figure Text Extraction Tool (FigTExT) to improve the performance of the OCR tool for figure text extraction through the use of three innovative components: *image preprocessing*, *character recognition*, and *text correction*. We first developed *image preprocessing* to enhance image quality and to improve text localization. Then we adapted the off-the-shelf OCR tool on the improved text localization for *character recognition*. Finally, we developed and evaluated a novel *text correction* framework by taking advantage of figure-specific lexicons.

**Results/Conclusions:**

The evaluation on 382 figures (9,643 figure texts in total) randomly selected from PubMed Central full-text articles shows that FigTExT performed with 84% precision, 98% recall, and 90% F1-score for text localization and with 62.5% precision, 51.0% recall and 56.2% F1-score for figure text extraction. When limiting figure texts to those judged by domain experts to be important content, FigTExT performed with 87.3% precision, 68.8% recall, and 77% F1-score. FigTExT significantly improved the performance of the off-the-shelf OCR tool we used, which on its own performed with 36.6% precision, 19.3% recall, and 25.3% F1-score for text extraction. In addition, our results show that FigTExT can extract texts that do not appear in figure captions or other associated text, further suggesting the potential utility of FigTExT for improving figure search.

## Introduction

Biomedical full-text articles incorporate a significant number of figures with such figures typically reporting experimental results, presenting research models, and providing examples of biomedical objects (e.g., cells, tissue, and organs). Figures represent important biomedical knowledge, and consequently figure mining has drawn much attention in the biomedical research community [1]–[8].

Most approaches to figure mining focus on extracting localization features from figures (e.g., [9]), figure classification ([1], [2]) and text-figure association [3], [10]–[13]. For example, the Subcellular Location Image Finder (SLIF) system [9] extracts information from fluorescence microscopy images and aligns image panels to their corresponding sub-legend. Shatkay et al. [14] integrated image features with text to enhance document classification. BioText [4] and Yale Image Finder [7] index figure legends and return figure+legend in response to a text query. We have also developed approaches for figure classification [2], [15], as well as natural language processing approaches for associating figure with text [3], figure summarization [11], [16] and figure ranking [13].

**Figure 1:**
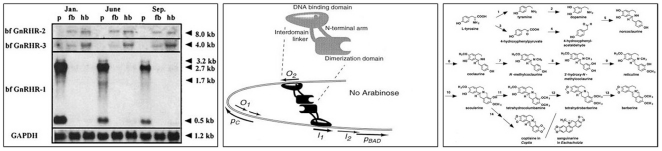
Examples of figure text in figures. Biomedical figures generally include biomedical named entities (e.g., tissue, species, chemical, and gene or protein names) and functional description (e.g., “DNA binding domain”). Biomedical figure text (i.e., text appearing in a biomedical figure) is important for understanding the meaning of a figure.

Biomedical figure text, that is, text appearing in biomedical figures is important for understanding the meaning of figures. However, few approaches have been developed for extracting text from figures. Figure 1 shows representative examples of biomedical figure text, including biomedical named entities (e.g., tissue, species, chemical, and gene or protein names) and function descriptions (e.g., “DNA binding domain”). Such examples show the potential value that figure text has for biomedical figure mining but also suggest some of the challenges of such work, which will be discussed below.

Existing work on text extraction from images has mainly focused on the open-domain of natural scene images [17], [18], [19] and videos [20]–[23] rather than biomedical figure text extraction. Previous research has applied off-the-shelf Optical Character Recognition (OCR) tools to figure retrieval [7] and figure panel detection [24]. Our own research has found that off-the-shelf OCR tools generally produce many recognition errors on biomedical figures; however, there is no published work on evaluating existing OCR tools for biomedical figures or improving the performance of such tools for biomedical work. Thus, this study is the first attempt for both tasks.

Figures are images. In the open domain, image text extraction is a relatively mature field and typically incorporates the following three steps: *text localization*, *character recognition*, and *text correction*. Open-domain off-the-shelf OCR tools can perform well [17], [18] under two conditions – that images are of high quality and that text is typically presented with a simple background. Unfortunately, both of these conditions are seldom met by biomedical figures; rather, we have observed that biomedical figures are frequently of low image quality and that the background of images tends to be complex. Furthermore, biomedical figures have domain-specific characteristics that include unexpected word boundaries (e.g., hyphens and other punctuation), domain-specific terms (e.g., gene and protein names), and symbols that do not appear in open-domain images. Therefore, we speculate that off-the-shelf OCR tools may not perform well on biomedical figures.

In this study, we first evaluated the performance of an off-the-shelf OCR tool. We then developed and evaluated a novel and domain-specific biomedical Figure Text Extraction Tool (FigTExT) for extracting text from biomedical figures. Thus, our study is an important step towards biomedical full-text mining.

## Methods

**Figure 2:**
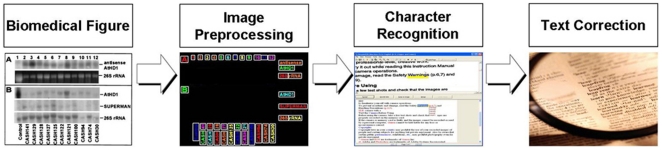
FigTExT (Figure Text Extraction Tool). FigTExT has three components: image preprocessing, character recognition, and text correction. Image preprocessing enhances not only text region detection by improving image contrast and determining the gray-level of figure text, but also image quality by up-sampling. FigTExT incorporates an off-the-shelf OCR tool for character recognition. For text correction, FigTExT first corrects misrecognized characters with a figure-specific lexicon and then refines the corrected result to filter out some spurious corrections.

As shown in Figure 2, FigTExT has three components: *image preprocessing*, *character recognition*, and *text correction*. *Image preprocessing* enhances not only text region detection by improving image contrast and determining the gray level of figure texts, but also image quality by up-sampling. FigTExT adapts an off-the-shelf OCR tool on the improved text localization for *character recognition*. For *text correction*, FigTExT first corrects misrecognized characters using a figure-specific lexicon and then refines the corrected result to filter out some spurious corrections.

### Image Preprocessing

#### A. Text Localization

Text localization detects text regions in images. In this study, we adapted Gatos et al.'s approach [19] to separate text regions from non-text regions because this approach has shown to perform well on high contrast text regions. However, the approach has to be repeated twice for both the given image and its inverted image because of the unknown gray-level of the figure texts. Therefore, for optimal text localization performance, we preprocessed figure images (i.e., using contrast enhancement and gray-level decision of figure texts) prior to separating text regions from the images.

**Figure 3:**
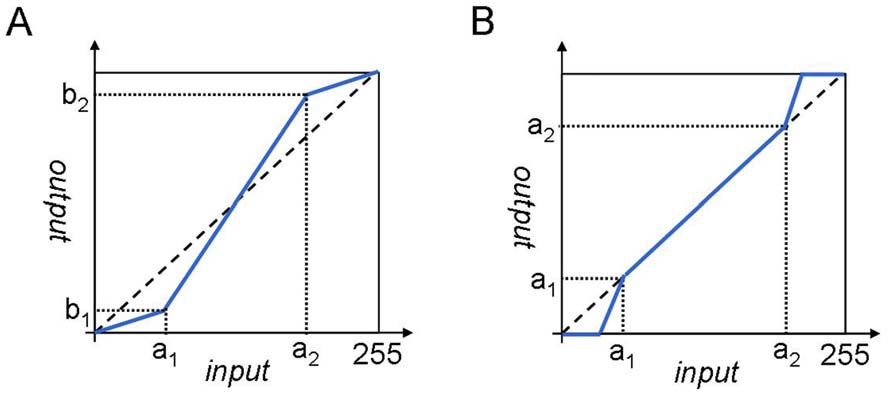
Contrast enhancement using modified contrast stretching transformation. (A) Conventional contrast stretching transformation may be able to enhance the contrast of non-text regions as well as text regions; therefore, false localization can be anticipated. (B) We focused on only enhancing the contrast of text regions, not non-text regions. Our figure data presented that the gray-level of black text is usually lower than 10, and that of white text is higher than 230 in the 256 gray-level image. Therefore, we modified the contrast-stretching transformation by setting a_1_ = 10 and a_2_ = 230 to lower and raise the gray-level of black text and white text, respectively, while preserving the contrast of non-text regions.

We developed the contrast stretching transformation [25] as shown in Figure 3(a) to enhance the contrast of figure texts. However, the transformation can enhance the contrast of both non-text regions and text regions, and as a result, may lead to false localizations. For this work, our strategy was to enhance only the contrast of text regions and ignore non-text regions. Since we found that the gray-level of black text in our figure data (256 gray-level images) was usually lower than 10, and that of the white text was higher than 230, we modified the contrast-stretching transformation by setting a_1_ = 10 and a_2_ = 230 to lower and raise the gray-level of black text and white text, respectively, while preserving the contrast of non-text regions, as shown in Figure 3(b).

To determine the gray-level of figure texts, we computed the average gray-level (*M*) of an input image (*I_O_*), as in Eq. (1). If *M* was higher than a certain threshold (*δ*), we considered the background image to be bright and the figure text dark, and we used the input image; otherwise, we inverted the input image before detecting text regions, as in Eq. (2). Implementing this approach enabled us to eliminate the redundancy of Gatos et al.'s approach.
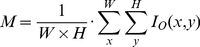
(1)

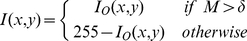
(2)


**Figure 4:**
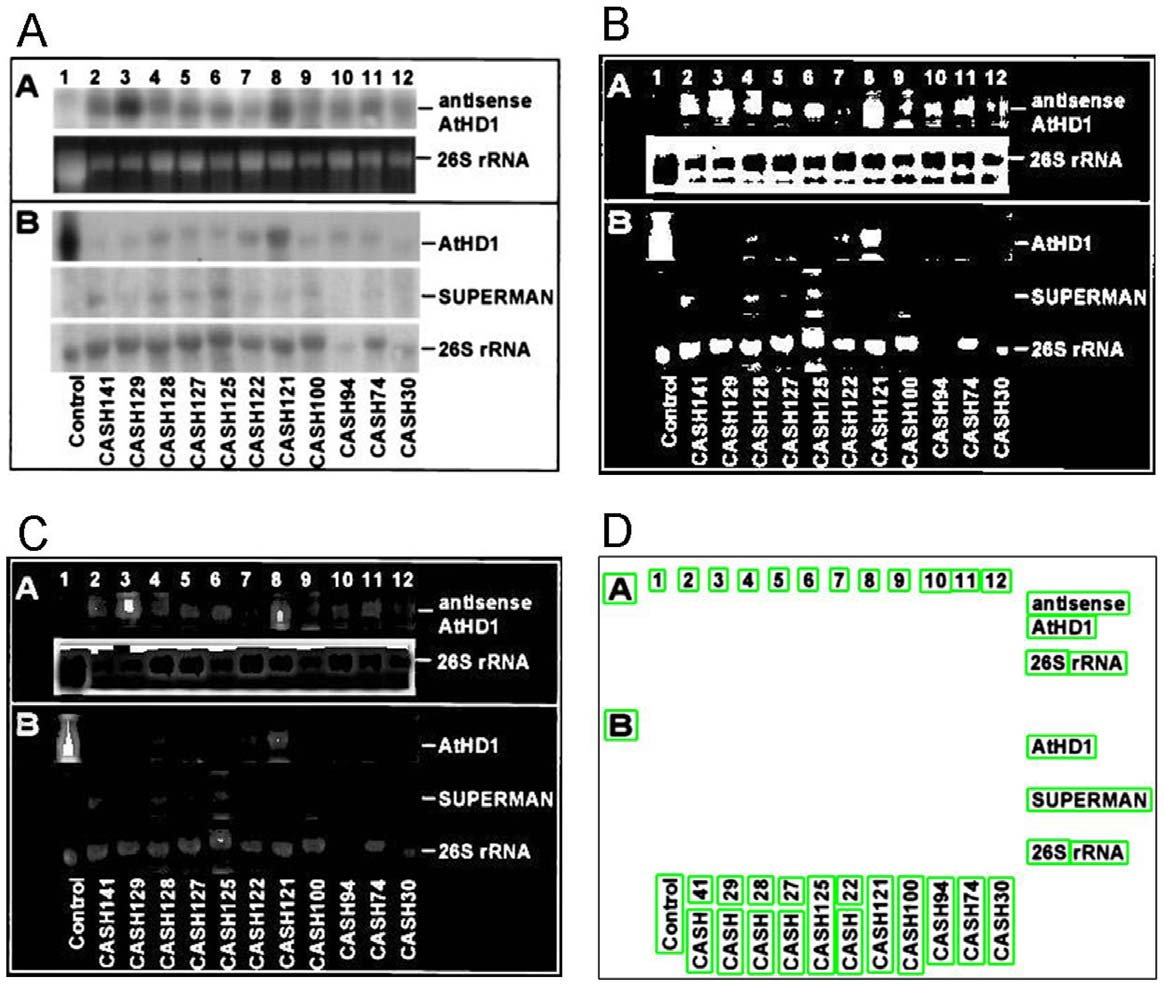
Figure text localization. (A) Input image. (B) Binary image. (C) Foreground objects. (D) Figure text regions.

Once the contrast was enhanced and the gray-level of the figure texts was determined, we adapted Gatos et al.'s approach to first obtain the binary image (Figure 4(b)) of the input image (Figure 4(a)) and then extract foreground objects (Figure 4(c)) according to the gray-level of the figure texts. Rather than identifying regions of foreground objects as others have done [17], [19], we extracted strong edges of foreground objects and then identified a set of connected components. This approach is motivated by the fact that figure text usually has a high contrast with its background due to the contrast stretching transformation in Figure 3(b). To detect character regions, we first applied geometrical constraints (e.g., size and aspect ratio of a character) to remove non-text regions. We then merged adjacent characters into the same text region with a morphological technique (Figure 4(d)). We first evaluated the performance of the text localization prior to applying it for FigTExT. To this end, we manually extracted 2,856 original text regions from 73 figure images randomly selected from the open-access articles deposited in PubMed Central. We then counted the number of correctly detected text regions (*N_c_*), the number of incorrectly detected text regions (*N_f_*), and the number of missed text regions (*N_m_*). Recall is computed as *N_c_*/(*N_c_*+*N_m_*); precision as *N_c_*/(*N_c_*+*N_f_*) and F1-score as the harmonic mean of recall and precision. Our evaluation results showed that our figure text localization attained approximately 84% precision, 98% recall, and 90% F1-score.

#### B. Image Up-sampling

As described earlier, off-the-shelf OCR tools generally perform well with high-quality images. In order to increase the quality of an image, we applied an up-sampling method called the bi-cubic interpolation method, which has shown to outperform other interpolation methods (e.g., nearest neighborhood and bilinear interpolation) [26].

### Character Recognition

After localizing figure text regions, we then applied the off-the-shelf OCR tool. In this study, we chose a widely used OCR tool, SimpleOCR API [27], for recognizing characters in the localized text regions. SimpleOCR used an English dictionary (77,537 words) for text correction. Prior to applying it for FigTExT, we first evaluated the performance of SimpleOCR API on high-quality document images which consist of 31,479 characters (including letters, numbers, and symbols). Our evaluation results showed that SimpleOCR API attained 97% in overall accuracy and that most errors were due to the misinterpretation of lowercase letters (e.g., ‘e’ and ‘m’) and symbols.

### Text Correction

Text correction is a well-studied field in the open domain. Dictionary-based approaches [28], [29], [30] correct typographic mistakes such as insertions, deletions, substitutions and transpositions of letters by replacing an error word token with its correct formation, typically a word in a lexicon. Similarity and frequency information have been used to rank candidate words using several approaches, including edit distance [31], [32], *n*-grams [33], [34], probabilistic model [35], and neural nets [36]. One challenge of dictionary-based methods is the computational time needed to examine candidate words in a large lexicon. To solve this problem, Lucas et al. [37] suggested reusing computation in a trie-formatted lexicon, and Schambach [38] eliminated words from consideration based on the low probability of their constituent characters.

In addition to dictionary-based approaches, context-based approaches have also been developed for text correction. Context-based approaches detect and correct words errors with contextual-similarity-based methods [39], [40], web knowledge-based methods [41], [42], probabilistic models [43], [44], [45], and latent semantic analysis [46]. One advantage of using context-based approaches is that the computation time is lower (although the training is costly). However, such context-based approaches depend on proper contexts, which are not always available [47].

Nearly all off-the-shelf OCR tools have a built-in spelling correction component using an open-domain dictionary for text correction. However, such an open-domain dictionary does not include domain-specific terms that are likely to be encountered in biomedical figure text, such as gene or protein names and cell or tissue types. We therefore developed an approach to post-correct characters wrongly recognized by the OCR tool with a figure-specific lexicon, to be described below.

#### A. Lexicon Construction

We developed different figure-specific lexicons and evaluated them for figure text recognition. Since figures are a part of full-text articles and the content of figures – including their important biomedical findings and methodologies – are usually described in the associated text (e.g., title, abstract, caption, or the full-text of the article in which a figure appears) [3], it is therefore reasonable to assume that figure text also appears in its surrounding context.

To test this hypothesis, we manually examined our figure collection (a collection of 382 figures, see “Data and Gold Standard” in the Methods section) and found that 26.8% of figure text appears in figure captions, 26.8% in figure-associated text, 34.4% in figure caption + associated text, and 42.2% in the full-text of the articles they accompany. We found that it is nearly impossible to build a lexicon that can recover 100% of figure text (for details, see Error Analysis). Accordingly, we built four figure-specific lexicons (caption, associated text, caption+associated text, full-text) and evaluated their performance for post-OCR text correction.

#### B. Text Correction

Biomedical figure text rarely takes the form of complete sentences; rather, such text generally consists of abbreviations, individual words, word fragments or phrases, as well as these in combination. Therefore, we speculated that context-based post-text correction methods [31], [32] would not work well for post-OCR text correlation and explored lexicon-based approaches.

Lexicon-based approaches require the identification of a specific lexicon (or word) as a text correction candidate. Such approaches match each recognized word (*w*) with each word (*c_i_*) in the lexicons and calculate the similarity between the two words. We explored three state-of-the-art word-similarity metrics for this work: edit distance (ED) [48], longest common sequence (LCS) [49], and multiple sequence alignment (MSA) [50].

**Figure 5:**
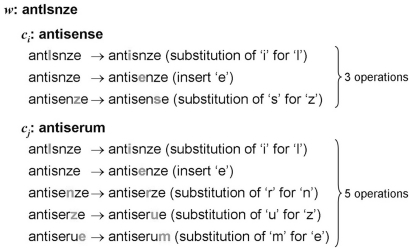
Example of edit distance (i.e., *Levenshtein* distance). Three edit operations (i.e., two substitutions and one insertion) are required to transform “antlsnze” to “antisense”, while five edit operations (i.e., four substitutions and one insertion) are required to transform “antlsnze” to “antiserum”. Therefore, according to ED, “antisense” has a higher similarity than “antiserum” to the recognized form “antlsnze”.

ED measures the minimum number of edit operations (i.e., insertion, deletion, or substitution of a single character) required to transform one word into another word: the lower an ED, the higher the similarity between two words. Figure 5 shows an example in which we applied ED to compute the number of edit operations between the recognized word, *w*="antlsnze”, and its candidates, *c_i_*="antisense” and *c_j_*="antiserum.” Three edit operations (i.e., two substitutions and one insertion) are required to transform “antlsnze” to “antisense,” while five edit operations (i.e., four substitutions and one insertion) are required to transform “antlsnze” to “antiserum.” Therefore, according to ED, “antisense” has a higher similarity than “antiserum” to the recognized form “antlsnze” and is thus more likely to be the original form.

LCS identifies the longest subsequence common to a set of words. A subsequence is a sequence that appears in the same relative order in all instances but not necessarily contiguously. For example, the LCS of the two words (*w* and *c_i_*) in Figure 5 is “antsne,” and the similarity of the two words is measured by the number of the letters in their LCS, which is 6, while the LCS of the two words (*w* and *c_j_*) is “ants”, and its similarity is 4. Therefore, LCS suggests that “antisense” is more likely than “antiserum” to have been the word incorrectly recognized as “antlsnze.”

**Figure 6:**
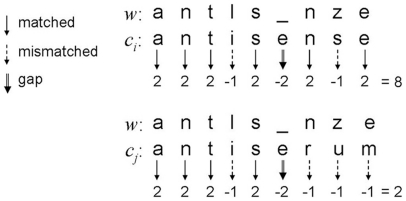
Example of multiple sequence alignment. The LCS of the two words (*w* and *c_i_*) is “antsne”, and its similarity is 6, i.e., the length of its LCS (“antsne”), while the LCS of the two words (*w* and *c_j_*) is “ants”, and its similarity is 4. Therefore, LCS suggests that “antisense” rather than “antiserum” is the correct form of “antlsnze.”

Similar to LCS, MSA also identifies regions of similarity between a word and a set of words. In contrast to LCS, however, it provides a gap penalty as well as match and mismatch scores to contribute to the overall score of alignments with a higher MSA score indicating a greater degree of similarity between the words. In this study, we assigned a positive match score (2), a negative mismatch score (−1), and a negative gap penalty (−2). Figure 6 shows an example in which MSA was used to compute the similarity of *w* with respect to *c_i_* and *c_j_* in Figure 5. As shown in Figure 6, there were 6 matching characters, 2 mismatched characters, and 1 gap between *w* and *c_i_*; thus, MSA provides a value of 8. On the other hand, there were 4 matching characters, 4 mismatched characters, and 1 gap between *w* and *c_j_*; thus, MSA provides a value of only 2. Therefore, similar to ED and LCS, MSA selects “antisense” as the original form misrecognized by OCR as “antlsnze.”

Although in this illustration, the three similarity metrics produce similar results for text correction, the three algorithms differ in many other cases. In this study, we evaluate all three algorithms for biomedical figure text correction.

#### C. Refinement of Text Correction

As described earlier, only 42.2% of figure texts appear in their associated full-text articles. Therefore, with our lexicon-based approach, 57.8% of figure texts that do not appear in the full-text article may be falsely 'corrected', even though some of them are correctly recognized by the off-the-shelf OCR tool. To overcome this problem, we developed an additional process to refine the result of text correction.

We assumed that if the recognized word (*w*) is misspelled, but its original word (w*_c_*) exists in the lexicon, there is a certain degree of overlap between two words. As a measure of this, we first parsed words into letter *n*-grams. During the parsing process, we included the “beginning” and “end” spaces surrounding the word [43]. We then estimated the number of matched *n*-grams between words (*ω_TF_*). Finally, the overlap (*T_overlap_*) between two words can be computed as
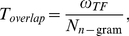
(3)where *N_n-_*_gram_ is the total number of letter *n*-grams of a recognized word. If *T_overlap_* is higher than a certain threshold (*γ*), the corrected word (w*_c_*) is acceptable, as in Eq. (4). Otherwise, the recognized word (*w*) is acceptable since it is considered as a wrong text correction.
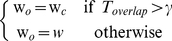
(4)where w*_o_* is the final result of FigTExT.

### Data and Gold Standard

The gold standard we used for developing and testing comprises 382 figures appearing in 70 full-text articles randomly selected from PubMed Central. We then manually transcribed 9,643 figure texts from the figure collection; this was done by both the first author of this paper and one University of Wisconsin college student. The two transcribers showed an agreement of 96% and a Cohen's kappa value of 0.95 with 95% confidence. After redundant figure texts were removed, there were 3,853 unique figure texts used for evaluation. We used 30% of our figure collection for developing and the remaining 70% for testing.

### Important Figure Texts

Not all figure texts are semantically rich. Some figure characters (e.g., panel labels) may be important for certain data-mining tasks (e.g., panel detection [24]), but those texts may not represent the semantics of the figures. On the other hand, certain figure texts (e.g., gene and protein names) may play an important role for representing the semantics of figures, and we evaluated FigTExT for identifying those semantically important figure texts.

First, we evaluated whether semantically important figure texts could be reliably annotated by domain experts. To this end, we randomly selected 60 figures in our figure collection and asked three domain experts (PhDs in the bioscience domain) to independently identify important figure texts. We calculated inter-rater agreement. We also evaluated FigTExT using the extracted important figure texts as a gold standard.

### Figure Texts that Do Not Appear in Lexicons

As stated earlier, 57.8% figure texts do not appear in the full-text. A system that can uncover those “lost” texts has the potential to improve figure search. We therefore evaluated the performance of FigTExT on those figure texts that do not appear in lexicons.

### Evaluation Methods

Figure text incorporates both word characters and other symbols. To simplify the evaluation, we ignored numbers and special symbols (e.g., +, −, @, #, %, etc.) and evaluated word characters only. Our evaluation was strict: a recognized text was considered as correct if every character and its character sequence completely matched the gold standard text. We then counted the number of recognized words (*N_R_*), the number of correctly recognized words (*N_C_*) of the recognized words, the number of transcribed figure texts in figures (*N_F_*), and the number of correctly retrieved words (*N_T_*) of transcribed figure texts. We adopted precision, recall, and F1-score as the evaluation metric. Precision is computed as *N_C_*/*N_R_*; recall as *N_T_*/*N_F_*; and F1-score as the harmonic mean of recall and precision.

## Results

### Image Preprocessing

As shown in Table 1, using the OCR tool alone attained only 36.6% precision, 19.3% recall, and 25.3% F1-score for figure text extraction. When text localization was applied prior to the application of the OCR tool, the performance was only slightly improved; this may be due to the fact that figure texts in the localized text regions were still of too poor a quality to be correctly recognized by the OCR tool. In contrast, when image up-sampling was applied prior to the application of the OCR tool, the performance improved, attaining 37.3% precision, 31.1% recall, and 33.9% F1-score, which was, respectively, 0.7%, 11.8%, and 8.6% (absolute value) higher than the performance of the OCR tool alone. Interestingly, when we integrated both text localization and image up-sampling – we applied text localization first and then added image up-sampling – both recall and F1-score values further increased by 24.8% and 10.8%, respectively (absolute value), attaining the final scores of 37.2% precision, 55.9% recall, and 44.7% F1-score, which is, respectively, 0.6%, 36.6%, and 19.4% (absolute value) higher than the results of applying the OCR tool alone.

**Table 1:** Results of text localization and up-sampling prior to the application of the OCR tool.

	Precision (%)	Recall (%)	F1-score (%)
Off-the-shelf OCR tool	36.6	19.3	25.3
Applying text localization prior to OCR tool	36.6	19.5	25.4
Applying image up-sampling prior to OCR tool	37.3	31.1	33.9
Applying text localization and image up-sampling prior to the OCR tool (baseline system)	37.2	55.9	44.7

### Text Correction

We evaluated text correction on three similarity metrics: ED, LCS and MSA, as well as on four figure-specific lexicons: figure caption, associated text, caption+associated text, and full-text. The average numbers of word tokens were 99, 410, 509, and 6,156, respectively, corresponding to the four lexicons. We found that text correction methods performed poorly without image preprocessing. As a result, our text correction methods were built upon the improved OCR tool, which integrates both the processes of text localization and image up-sampling described in the previous paragraph (Image Preprocessing).

As shown in Table 2, of all four figure-specific lexicons, ED outperformed both LCS and MSA, and MSA outperformed LCS. With figure captions, for example, the performances of ED, LCS, and MSA were 48.2%, 27.4%, and 38.1% F1-score, respectively.

**Table 2:** Performance of FigTExT for four figure-specific lexicons.

	Figure caption	Associated text	Caption+Associated text	Full-text article
	F1-score (%) (Recall, Precision)	F1-score (%) (Recall, Precision)	F1-score (%) (Recall, Precision)	F1-score (%) (Recall, Precision)
ED	48.2 (40.6, 59.4)	47.0 (41.8, 53.7)	56.2 (51.0, 62.5)	51.6 (56.1, 47.8)
LCS	27.4 (28.5, 26.4)	24.3 (26.4, 22.4)	30.8 (34.2, 28.0)	18.7 (24.4, 15.1)
MSA	38.1 (37.2, 38.6)	36.1 (37.7, 34.6)	42.9 (46.7, 39.6)	32.1 (41.3, 26.3)

Of the four types of lexicons, caption+associated text outperformed all three other lexicons in all three similarity metrics (ED, LCS and MSA), attaining the best F1-score of 56.2% in the ED method, followed by F1-scores of 30.8% and 42.9% F1-scores using the LCS and MSA methods, respectively. In contrast, the results of using figure caption, associated text, and full-text article as lexicons are mixed. For example, using full-text articles as the lexicon, the ED method led the performance of 51.6% F1-score, outperforming figure caption and associated text. On the other hand, using full-text as the lexicon did not lead to good performance for LCS and MSA, results in F1-scores of 18.7% and 32.1%, respectively. Figure caption outperformed both associated text and full-text when LCS and MSA were applied, attaining an F1-score of 27.4% and 38.1%, respectively.

As described earlier, we developed methods in text correction refinement to prevent inaccurate out-of-lexicon text correction. As shown in Table 3, the refinement approaches increased the performance of LCS and MSA. On the other hand, the performance of ED decreased, although it still outperformed LCS and MSA. Similar to the results shown in Table 2, caption+associated text remained as the best performing lexicon.

**Table 3:** Performance of FigTExT for four figure-specific lexicons with refinement.

	Figure caption	Associated text	Caption+Associated text	Full-text article
	F1-score (%) (Recall, Precision)	F1-score (%) (Recall, Precision)	F1-score (%) (Recall, Precision)	F1-score (%) (Recall, Precision)
ED	47.2 (58.2, 39.8)	47.7 (58.5, 40.3)	49.1 (60.6, 41.3)	49.4 (61.4, 41.3)
LCS	45.8 (56.2, 38.6)	45.2 (55.2, 38.3)	46.4 (56.9, 39.1)	44.1 (54.1, 37.2)
MSA	46.6 (57.5, 39.2)	46.7 (57.6, 39.3)	47.9 (59.3, 40.2)	46.8 (58.1, 39.1)

### Performance in Terms of Character and Term Accuracies

**Figure 7:**
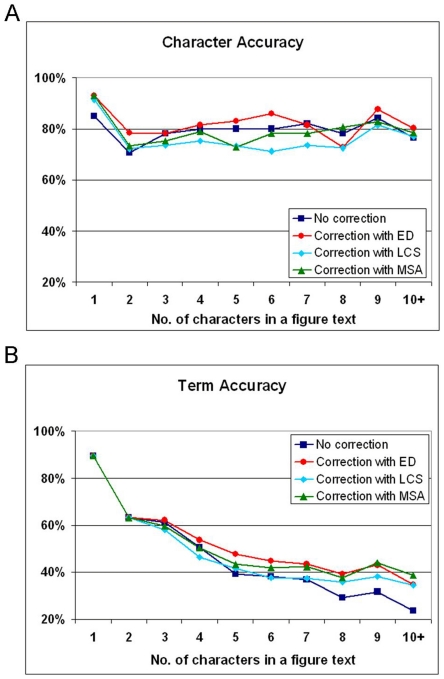
Performance of FigTExT in terms of character and term accuracies. (A) Overall character accuracy of the baseline system (i.e., No correction) is 79.2% and its variance 0.9%. Our character recognition performed equally well regardless of word length. ED attained 81.7% overall character accuracy, which is 2.5% higher than the baseline system. In contrast to ED, LCS and MSA attained 75.5% and 78.8% overall character accuracies, which is 3.7% and 0.4% lower, respectively, than the baseline system. (B) Term accuracy decreases when the number of characters in a figure text is increased. ED still outperformed both LCS and MSA, and MSA outperformed LCS in all word lengths.

We evaluated whether the performance of FigTExT related to word length. Figure 7(a) plots character accuracy as a function of word length. The plot is based on the best system (ED+caption+associated text) in Table 3 because it has the highest recall. As shown in Figure 7(a), the overall character accuracy of the baseline system (i.e., No correction in Figure 7(a)) was 79.2% and its variance 0.9%. The results show that FigTeXT's performance does not depend on word length. ED attained 81.7% overall character accuracy, which is 2.5% higher than the baseline system. In contrast to ED, LCS and MSA attained 75.5% and 78.8% overall character accuracies, which is 3.7% and 0.4% lower, respectively, than the baseline system.

Figure 7(b) shows the word accuracy (in contrast to character accuracy) of FigTExT. As expected, word accuracy decreases when the number of characters in a figure text is increased. ED still outperformed both LCS and MSA, and MSA outperformed LCS in all word length.

### FigTExT on Important Figure Texts

Table 4 shows the results of inter-rater agreement on identifying important figure texts. The pairwise agreement of the three annotators A, B, and C showed a kappa value of 0.911, 0.936 and 0.563 for *A* and *B*, *B* and *C,* and *A* and *C,* respectively. The lower agreement between *A* and *C* was due to *C* selecting much more important figure texts.

**Table 4:** Inter-rater agreement and its kappa value on important figure text (95% confidence).

Pair of annotators	Agreement	Cohen's kappa value
*A* − *B*	96.5%	0.911
*B* − *C*	98.0%	0.936
*A* − *C*	80.8%	0.563

With the best FigTExT system (ED+caption+associated text) as shown in Table 2, we evaluated the system on important figure texts. As shown in Table 5, the joint *A* and *B* data led to the highest number of figure texts comparing to other pairs. However, its precision, recall, and F1-score presented the lowest values. In contrast, the joint *A* and *C* text sets resulted in the lowest number of figure texts and resulted in the best performance: an F1-score of 77%. The three domain experts annotated a combined set of 757 important figure texts, for which FigTExT performed with 73.0% precision, 62.0% recall and 67.1% F1-score. We found that 69.2% of the 757 important figure texts appeared in the lexicon (caption+associated text), the percentage of which is significantly higher than the 34.8% of all figure texts that appear in the lexicon.

**Table 5:** Performance of FigTExT on the important figure texts identified by annotators.

	*A*∩*B*	*B*∩*C*	*A*∩*C*	*A*∪*B*∪*C*	All figure texts
No. of figure texts	78	65	47	757	3,853
Precision (%)	37.2	74.3	87.3	73.0	62.5
Recall (%)	44.7	52.6	68.8	62.0	51.0
F1-score (%)	40.6	61.6	77.0	67.1	56.2

For the remaining 30.8% of important figure texts not appeared in the lexicon, FigTExT did not extract any original texts since they were corrected with word tokens in the lexicon. However, after we applied the text correction refinement, FigTExT recovered 38.1% figure texts that do not appear in the lexicons, although the overall FigTExT's precision was reduced from 40.1% to 24.3%.

### Error Analysis

Our results show that only 42.2% of figure texts appear in the corresponding full-text articles, the result of which explained the low recall of FigTExT. We manually analyzed why figure texts do not appear in the full-text.

**Figure 8:**
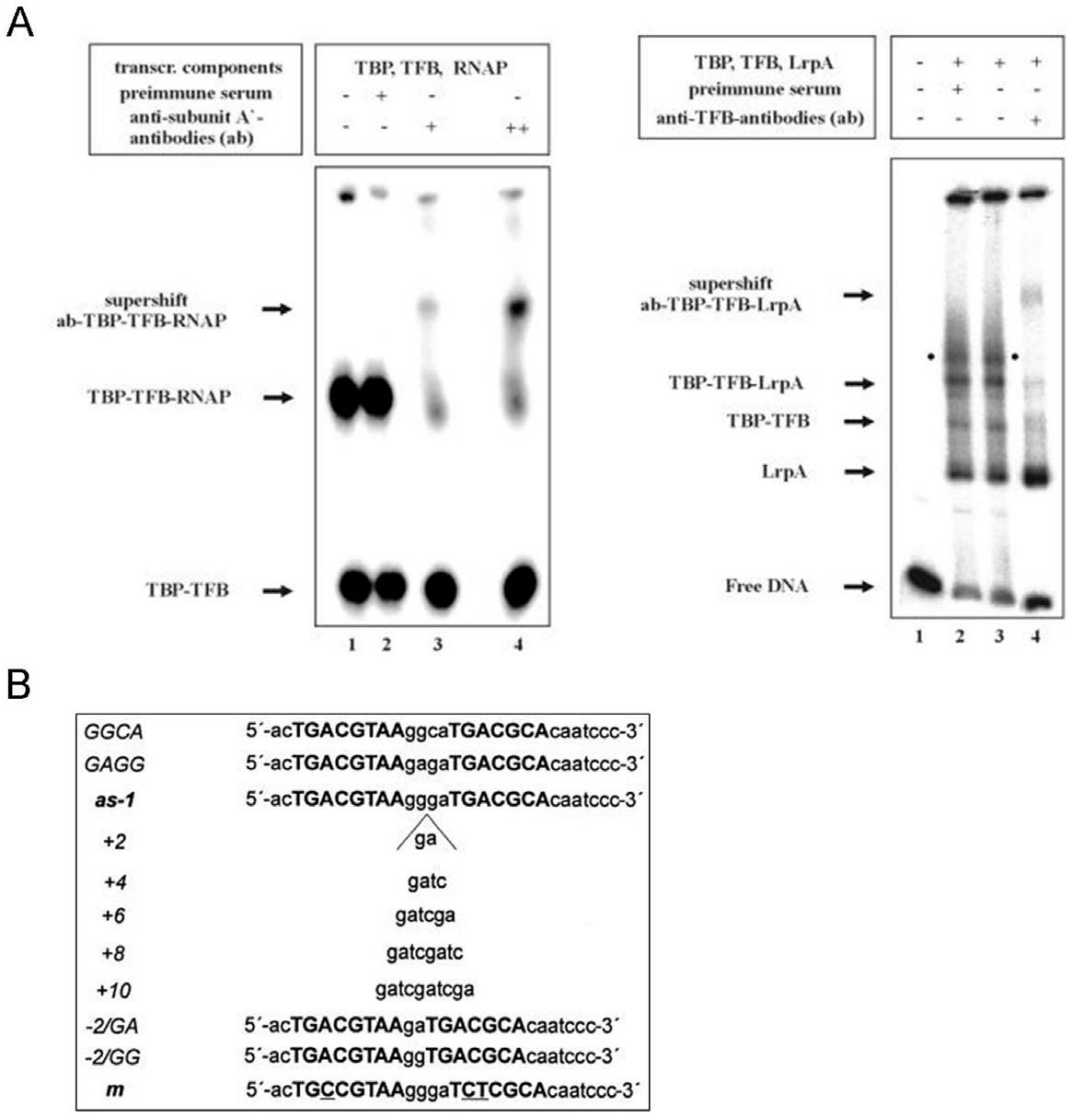
Reasons that figure text do not appear in the lexicons. (A) Abbreviations and linked terms. (B) Gene sequences.

1. **Abbreviations. **Biomedical researchers tend to maximize the usage of image space and using abbreviations is one strategy. We found that abbreviations frequently appear in figures. For example, as shown in Figure 8(a), “transcr.” is the abbreviation of “transcription” and “ab” is that of “antibodies”. However, many abbreviations that appear in figures do not appear in the full-text article, and this constitutes a challenge.
2. **Linked Terms.** Biomedical researchers are creative in their use of limited image space. We found that two or more different terms were connected by symbols such as ‘–’, ‘+’, and ‘/’. For example, as shown in Figure 8(a), “TBP-TFB-RNAP” is shown in the full-text as “the association of RNAP to the TBP–TFB complex” and “TBP-TFB-LrpA” stands for “the binding sites of LrpA and TBP/TFB”.
3. **Gene Sequence.** We found that figures frequently incorporate gene sequences, many of which do not appear in the full-text article. For instance, as shown in Figure 8(b), of the three sequences, “GGCA” is the only one that appears in the full-text.

We analyzed sources of errors when figure texts appeared in the full-text. Using the best system (ED+caption+associated text in Table 2) as FigTExT, our results show that 62.4% of figure texts were correctly identified. None of the figure texts not appearing in the lexicon were extracted since they were corrected with word tokens in the lexicon. Our manual analyses of the remaining 37.6% of wrongly identified figure texts revealed the following five additional causes of errors: complexity, thick stroke, contrast, font size, and font type.

**Figure 9:**
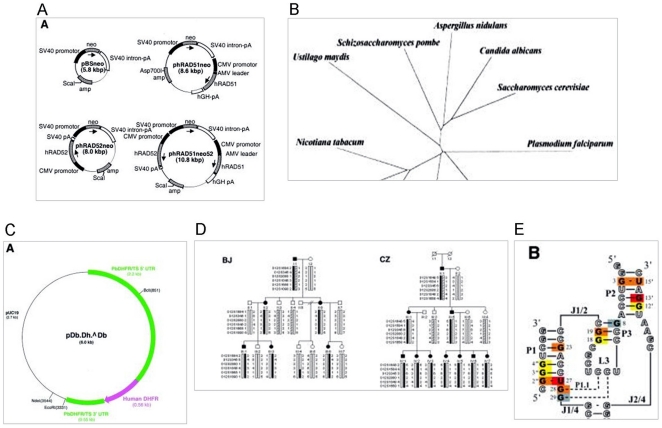
Additional reasons for OCR errors. (A) High image complexity. (B) Thick stroke. (C) Low image contrast. (D) Small font size. (E) Non-standard font type.

**4) High Image Complexity.**Biomedical figures are complex. Text and image content are frequently intertwined (an example is shown in Figure 9(a)), and as a consequence, text localization frequently detects non-text regions by mistake and decreased both the recall and precision.
**5) Thick Stroke.** Thick strokes not only close the loops in letters such as “a” and “e”, completely or partially, but they also often touch neighboring characters, as shown in Figure 9(b). This sometimes makes it difficult even for human to correctly identify such figure texts. As a result, character recognition and text correction can produce errors even when text localization correctly detects text regions.
**6) Low Image Contrast. **Image contrast is as important as image quality for text recognition. Color text shown in Figure 9(c) usually presents visually high contrast with background. However, its gray-level difference is much lower than that of black text. This low contrast prevents FigTExT from localizing text regions and consequently from recognizing text correctly.
**7) Small Font Size. **In general, figures have limited space for incorporating figure text. Hence, authors often use a small font size when inserting text. Small font size, however, often lowers both image quality and contrast, as in Figure 9(d), serving as another error source despite enlarging it using bicubic interpolation.
**8) Non-Standard Font Type. **Typically, the off-the-shelf OCR tool that we used in this study can recognize such standard font types as Arial, Century, and Times New Roman. However, we found that authors often use non-standard font type (e.g., outlined) to emphasize their results (e.g. Figure 9(e)). Although text localization can detect these non-standard font-type character regions, the OCR tool cannot always deal with them properly.

## Discussion

Although the off-the-shelf OCR tool attained 97% accuracy in character recognition for high-quality document images, our results (as shown in Table 1) show that it performed poorly (25.3% F1-score) on biomedical figures. Therefore, it is important to develop a recognition tool specifically for biomedical figure text. Our FigTExT was built by implementing components for image preprocessing, character recognition, and text correction. Below, we will discuss each component.

### Image Preprocessing

We explored figure text localization and image up-sampling techniques for image preprocessing. Our results show that figure text localization did not affect the performance of the OCR tool in spite of its high performance (90% F1-score). Meanwhile, image up-sampling improved the performance of the OCR tool to attain a 33.7% F1-score. Accordingly, image up-sampling is more effective than figure text localization for biomedical figures. We speculate that poor image quality was accountable for the performance difference between the two approaches. Our integrated approach takes advantage of figure text localization for removing nontext regions and image up-sampling for improving the quality of localized figure texts. As a result, the performance of the integrated approach significantly improved, attaining a 44.7% F1-score, as shown in Table 1.

### Text Correction Methods

We explored three different similarity metrics – edit distance (ED), longest common subsequence (LCS), and multiple sequence alignment (MSA) – and the results show that ED performed the best (as shown in Tables 2 and 3). In contrast to ED, both MSA and LCS are approximation matching algorithms that did not work well in figure text correction. For example, the off-the-shelf OCR tool misrecognized a protein “Rad52p” as “Radsap”. ED corrected it as “Rad52p”, while LCS corrected it as “paraformaldehyde/saponin” because all characters in “Radsap” appeared in “pa**ra**formal**d**ehyde/**sap**onin”. Since MSA added a penalty (negative) to the overall score in mismatch and therefore it performed better than LCS.

We also explored text correction refinement based on letter *n*-gram term frequency, and our results show that the approach did not work well in biomedical figures. On the other hand, although the overall F1-scores did not improve, the best recall increased from 51% to 60.6%, indicating that the refinement approaches may still be useful if a user prefers a high recall.

### Figure-Specific Lexicons

One significant challenge for biomedical figure text extraction is that figure texts are domain-specific and include specialized terms (e.g., gene or protein names), unexpected word boundaries (e.g., hyphens and other punctuation), abbreviations, etc. For instance, an ordinary dictionary includes “DNA” and “RNA”, but it does not include “rDNA” and “rRNA” since they are specific types of “DNA” and “RNA”. As a result, off-the-shelf OCR tools do not perform well on biomedical text, and we therefore constructed domain-specific lexicons.

We show that domain-specific lexicons improve the performance of FigTExT. We evaluated four domain-specific lexicons: figure caption, figure associated text, figure caption+associated text, and full-text. Our results show that without domain-specific lexicons, FigTExT attained a 44.7% F1-score. Adding captions and associated text improved F1-scores to 48.2% and 47%, respectively. The addition of caption+associated text further improved the F1-score to 56.2%. Interestingly, when the full-text article was used as the lexicon, the performance decreased.

A full-text article typically has over 6,000 word tokens and therefore may introduce “noise.” For example, we found that our character recognition system misrecognized the figure text “serum” as “seeqmz.” Our text correction system matched “seeqmz” with the lexicon. When the full-text was used as the lexicon, the word “seems” was selected because it had a lower ED (one deletion and one substitution) than “serum” which requires two substitutions and one deletion. In contrast, the error did not occur when the lexicon was caption+associated text. These results show that bigger does not necessarily mean better.

Our domain-specific lexicons have limitations. As shown in the error analysis, only 42.2% of our figure texts appeared in the corresponding full-text articles, which significantly reduced the recall of the overall FigTExT system. In contrast, 69.2% of important figure texts appeared in the lexicon – a significant increase of 34.8% (absolute value) – and it is not surprising that FigTExT attained 73% precision, 62% recall, and 67.1% F1-score, which is 10.5%, 11%, and 10.9% (absolute value), respectively, for detecting important figure texts. The performance was significantly better than the performance of FigTExT on all figure texts (Table 2). This result suggests a positive correlation between the coverage of a lexicon and FigTExT's performance.

### Conclusion and Future Work

In this study, we reported the development of FigTExT (Figure Text Extraction Tool), a domain-specific image-natural language processing system that automatically extracts text from biomedical figures. As a part of the development of FigTExT, we explored figure text localization and image up-sampling, which improved the performance of an off-the-shelf OCR tool. In addition, we developed approaches for text correction in which we explored different domain specific lexicons and similarity metrics. In addition, we explored domain-specific text-correction refinement.

Our study is an important step towards biomedical full-text mining. Since we found that FigTExT's performance is mostly positively correlated with the coverage of figure texts in domain-specific lexicons, in future work we will explore approaches to increase the coverage of lexicons. We may do so by adding words that appear in related articles to the lexicon.

However, our results also show that lexicon coverage was not always positively correlated with FigTExT's performance. The best FigTExT system incorporated caption+associated text as the lexicon, outperforming the system that incorporated the larger full-text as the lexicon. Lexicon quality is also important. Therefore, we will explore natural language processing approaches to improve the quality of lexicons. For example, as a part of these approaches, we will find ways to limit lexicons to domain-specific named entities including gene, protein, small molecules, tissue names, etc. We will also explore approaches by which abbreviations can be mapped to full-forms and then added to lexicons.

Another research direction we intend to pursue is that of image quality assessment. Since biomedical figures tend to be of low quality, an alternative is to extract from high quality images and figure texts only. We will also explore techniques implementing super-resolution [51], [52] to improve image quality.
